# Patient with Acute Coronary Syndrome in the Setting of an Extremely Rare form of Complex Congenital Anomalous Coronaries

**DOI:** 10.1055/s-0041-1729852

**Published:** 2021-10-18

**Authors:** Mahmoud Abdelnabi, Fady Gerges, Yehia Saleh, Eman Elsharkawy, Mohamed Sanhoury, Mahmoud Hassanein, Abdallah Almaghraby

**Affiliations:** 1 Clinical and Experimental Internal Medicine Department, Cardiology and Angiology Unit, Medical Research Institute, Alexandria University, Alexandria, Egypt; 2 Department of Cardiovascular Science, Mediclinic Al Ain Hospital, Al Ain, United Arab Emirates; 3 Department of Cardiology, Houston Methodist Hospital, Houston, Texas, United Sates; 4 Department of Cardiology, Faculty of Medicine, Alexandria University, Alexandria, Egypt

**Keywords:** anomalous single coronary artery, coronary angiography, dual left anterior descending artery, non-ST elevation acute coronary syndrome, coronary anomalies

## Abstract

A single coronary artery is an exceedingly rare anomaly. Hereby, we present an unusual case of a young patient with an acute coronary syndrome who was found to have a single coronary artery originating from a single ostium in the right sinus of Valsalva with dual left anterior descending (LAD) arteries arising from the right coronary artery with two different anatomical courses, and additionally one of those LADs running a malignant intra-arterial course.

## Introduction

In a single coronary artery (SCA), the entire coronary tree arises as a single trunk from the ascending aorta and no evidence of a second coronary artery is found. 1

Dual left anterior descending (LAD) artery was first described by Spindola-Franco et al, 2 in 1983. Based on conventional coronary angiography and computerized tomographic (CT) angiography data, the prevalence of dual LAD is estimated to be 1 and 4%, respectively. 3

## Case Presentation

A 38-year-old female patient with no cardiovascular morbidities presented to the emergency department with chief complaint of acute central chest pain that started 3 hours earlier and worsened in the last hour before coming to hospital. Patient had normal vital signs and unremarkable physical examination. Her 12-lead electrocardiogram (ECG) showed 0.5 mm ST segment depression in the left-sided limb and lateral precordial leads (I, aVL, V5, and V6). High-sensitive cardiac troponin I (hs-cTnI) was slightly positive at 0.12 ng/mL (normal range <0.04 ng/mL). She received a loading dose of dual antiplatelet (DAPT) agents and was planned for elective coronary angiography (CAG) during the same hospital admission. She was categorized as a case of non-ST elevation acute coronary syndrome. A bedside transthoracic echocardiography showed no regional wall motion abnormalities and an ejection fraction of 60% with no valvular abnormalities.

**Fig. 1:**
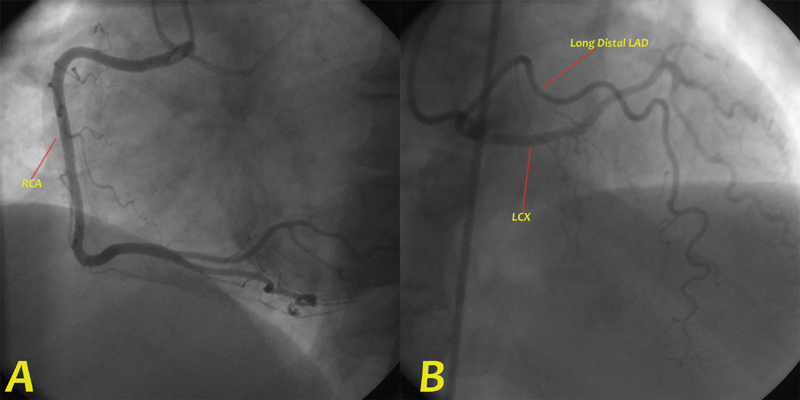
Coronary angiographic image showing single coronary artery (SCA) arising from the right sinus of Valsalva as a short common trunk which divides into ( **A** ) right coronary artery (RCA) that courses normally and bifurcates distally into posterior descending artery and posterolateral ventricular artery. ( **B** ) Both long distal left anterior descending (LAD) artery and left circumflex (LCX) artery originate separately from a common origin of SCA from the right sinus of Valsalva.

CAG through the right femoral artery unexpectedly demonstrated a SCA branching from the right coronary sinus (RCS). There was no left coronary system arising from the corresponding left coronary sinus. The right coronary artery (RCA) was normal in course and anatomy with large caliber, dividing into posterior descending artery and right posterolateral branches. The left system originated separately from the RCS, giving rise to a small left circumflex artery and two left anterior descending arteries with no obstructive disease along their course ( Fig. 1 ; Video 1 [available in the online version])

**Video 1**
Coronary angiography of a single coronary artery arising from the right sinus of Valsalva subsequently divided to normal right coronary artery and both long distal left anterior descending artery and left circumflex artery originating separately from a common origin of SCA from right sinus of Valsalva.

**Fig. 2:**
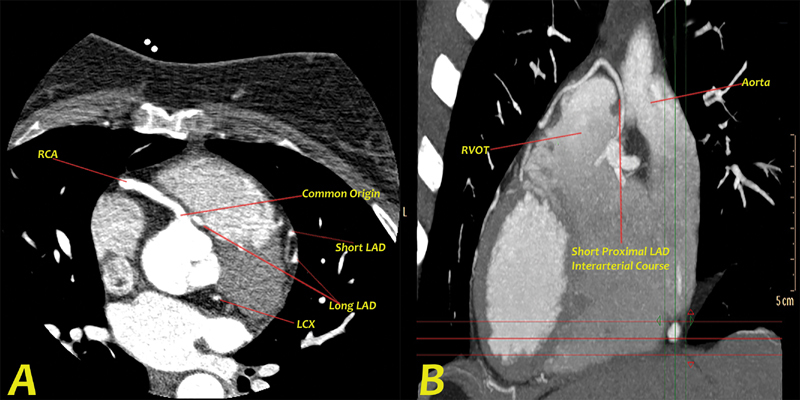
Computed tomography coronary angiography showing ( **A** ) axial view depicting a common coronary origin from the right sinus of Valsalava branching into right coronary artery (RCA) and long distal left anterior descending (LAD). Short axis of the short proximal LAD, long distal LAD and left anterior descending (LCX) arteries can be seen as well. ( **B** ) Maximum intensity projection images in the sagittal view depicting the interarterial course of the short proximal LAD between the aortic root and right ventricular outflow tract (RVOT).

**Fig. 3:**
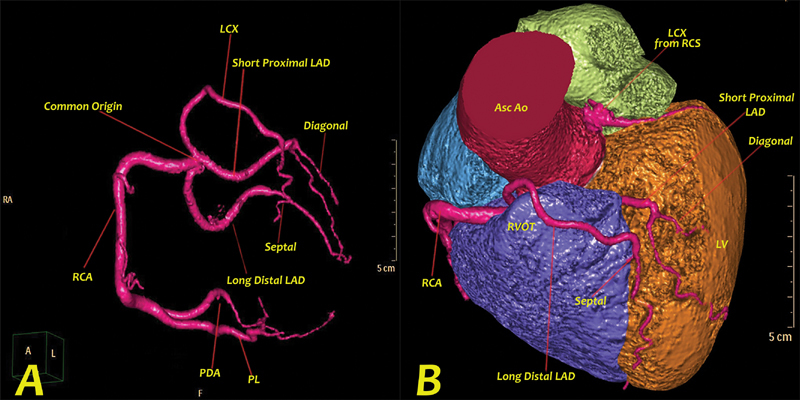
Computed tomography (CT) coronary angiography colored three-dimensional volume rendered CT angiography images showing ( **A** ) single coronary artery (SCA) arising as a common trunk from the right sinus of Valsalva and bifurcating into the right coronary artery (RCA) branching distally into posterior descending artery (PDA) and posterolateral ventricular (PLV) artery. A long distal left anterior descending (LAD) artery giving a small septal branch then coursing toward the left ventricular apex. A short proximal LAD artery giving a small diagonal branch and left circumflex (LCX) artery altogether with the long distal LAD originating separately from the SCA. ( **B** ) Left anterior oblique cranial view with the pulmonary trunk removed demonstrating the dual LAD anomaly with the short proximal LAD branching from the SCA from right sinus of Valsalva and passing between the right ventricular outflow tract (RVOT) and the aortic root taking a malignant interarterial course. Asc Ao, ascending aorta; PL, posterolateral; RCS, right coronary sinus.

Coronary computed tomography angiography (CCTA) was done to further characterize the course of the anomalous SCA, as well as to delineate the type of surgery indicated. This confirmed our diagnosis and demonstrated a benign course of the first proximal LAD artery, but a malignant course was to be distal long LAD that was running between the aorta and the right ventricular outflow tract ( Figs. 2 and 3 ; Video 2 [available in the online version]).

**Video 2**
Computed tomography coronary angiography of a single coronary artery arising as a common trunk from the right sinus of Valsalva.

After a heart team assessment, the patient was counselled to perform coronary artery bypass graft (CABG) surgery with reimplantation of the anomalous coronaries. However, she declined any surgical intervention, and she was treated conservatively with DAPT, B-blocker and statins followed by aspirin for life.

On frequent follow-up visits for the following 18 months, the patient was well, with no angina or effort intolerance.

## Discussion

Coronary anomalies are one of the most common cardiovascular causes of sudden death in young patients. 4

CCTA can precisely delineate the course of the anomalous artery and provide three-dimensional (3D) information about the relation of the anomalous artery to other cardiovascular structures, namely, cardiac chambers and major arteries. 5

Dual LAD anomalies have been classified into six different types based on the origin and course of the long LAD ( Table 1 ). 6 In types I, II, and III, both the long and the short LADs originate from the proximal LAD. In types IV, V, and VI, the long LAD originates from the proximal RCA or from the RCS. In types IV and V, the long LAD takes either an epicardial course or an intramyocardial course within the septal crest, while in type VI, which has recently been described by Maroney and Klein, 6 the long LAD courses between the right ventricular outflow tract (RVOT) and the aortic root. Type-VI dual LAD anomaly may have greater clinical significance than other types because compression of the coronary artery between the RVOT and the aortic root in situations of increased pulmonary blood flow could cause coronary blood flow restriction and sudden cardiac death. 7

**Table 1:** Classification of dual left anterior descending coronary artery

Type	Short LAD		Long LAD		Origin of major diagonal vessels
	Origin	Course	Origin	Course	
**I**	Proximal LAD	Proximal AIVG	Proximal LAD	Epicardial course on the left ventricular side of the proximal AIVG, reentering the distal AIVG	Proximal LAD and/or long LAD
**II**	Proximal LAD	Proximal AIVG	Proximal LAD	Epicardial course on the right ventricular side of the proximal AIVG, reentering the distal AIVG	Proximal LAD
**III**	Proximal LAD	Proximal AIVG	Proximal LAD	Intramyocardial course in the proximal septum, then either (i) emerging epicardially in the distal AIVG, or (ii) terminating intramyocardially as septal perforator arteries	Proximal LAD or short LAD
**IV**	LMCA	Proximal AIVG	RCA	(i) Epicardial free wall course anterior to the infundibulum of the RV traversing to the distal AIVG, or (ii) intramyocardial course within the septal crest emerging epicardially in the distal AIVG	Short LAD
**V**	LCS	Proximal AIVG	RCS	Intramyocardial course within the septal crest emerging epicardially in the distal AIVG	Short LAD
**VI**	LMCA	Proximal AIVG	RCA	Underneath the RVOT in the area of the interventricular septum	Short LAD

AIVG, anterior interventricular groove; LAD, left anterior descending artery; LCS, left coronary sinus; LMCA, left main coronary artery; Proximal LAD, the portion of the LAD just after bifurcation of the left main coronary artery into the LAD and left circumflex; RCA, right coronary artery; RCS, right coronary sinus; RV, right ventricle; RVOT, right ventricular outflow tract.

Recognition of anatomic variants of dual LAD anatomy is crucial for correct identification of these vessels during surgery and angiographic coronary interventions for coronary artery disease. 2 3

In an autopsy study among 126 military recruits, an anomalous coronary artery was responsible for one-third (21 of 64) of the cardiac deaths. In each case, the left coronary artery arose from the right sinus of Valsalva, coursing between the aorta and the pulmonary artery, leading to sudden cardiac death. 4

Surgical interventions include reimplantation of the anomalous artery to the aorta, osteoplasty, CABG of the anomalous artery, and pulmonary artery translocation. 8

In our patient, one of the two LAD arteries ran a malignant interarterial course, resulting in a presentation of ACS, however, with no clear coronary stenosis. Unfortunately, our patient was not willing to undergo surgery.

It is difficult to determine the course of an anomalous SCA using angiography alone. The 3D-CT images are extremely useful in demonstrating the dual LAD anatomy, as depicted in our case.
